# Case Report: Anti-flotillin 1/2 Autoantibody-Associated Atypical Dementia

**DOI:** 10.3389/fpsyt.2021.626121

**Published:** 2021-06-15

**Authors:** Niels Hansen, Claudia Bartels, Winfried Stöcker, Jens Wiltfang, Charles Timäus

**Affiliations:** ^1^Department of Psychiatry and Psychotherapy, University Medical Center Göttingen, Göttingen, Germany; ^2^Euroimmun Reference Laboratory, Lübeck, Germany; ^3^Neurosciences and Signaling Group, Department of Medical Sciences, Institute of Biomedicine (iBiMED), University of Aveiro, Aveiro, Portugal; ^4^German Center for Neurodegenerative Diseases (DZNE), Göttingen, Germany

**Keywords:** autoimmunity, autoantibodies, flotillin 1/2, dementia, phosphorylated tau protein

## Abstract

Flotillin proteins are involved in neurodegeneration and T-cell immunity. Here, we report the case of 65-year-old woman who presented with dementia, depressive symptoms, and a patient history involving speech problems. As diagnostics methods we applied magnetic resonance imaging, clinical examination, extensive neuropsychological testing, and cerebrospinal fluid analysis. Neuropsychological testing revealed major cognitive decline in attentional, executive, and memory functions together with impaired activities of daily living. The cerebrospinal fluid showed elevated phosphorylated tau protein 181. We identified serum autoantibodies against the flotillin 1/2 complex. Immunotherapy entailing four cycles of high-dose steroids resulted in less cognitive dysfunction along with reduced depressive symptoms in the second follow-up after starting steroids. In conclusion: probable autoimmune-mediated dementia associated with anti-flotillin 1/2 complex autoantibodies expands the phenotypic spectrum of anti-flotillin 1/2 antibody disease.

## Introduction

Neural autoantibodies have been detected in patients with atypical dementia (1, 2) and cognitive impairment (3). The term autoimmune dementia was recently coined by Flanagan et al. (4) and actual guidelines have been published recently (5). Till now, 11 different subforms of cell-surface autoantibody and 13 subtypes of intracellular antibody-related autoimmune dementia have been reported in a review encompassing an often early-onset or young-onset atypical dementia with a subacute onset and progressive time course (5). Here, we report for the first time about a 65-year-old woman presenting with a dementia combined with serum autoantibodies against flotillin-1/2 complex connected mainly to the plasma membrane surface. Flotillin proteins are assumed to be crucial players in autoimmune mediated encephalomyelitis (6), in T-cell immunity and activation (7, 8) as well as neurodegenerative diseases by accumulating in neurons (9). Flotillin proteins thus seem to be a key interface between autoimmunity and neurodegeneration. Autoantibodies might interfere with the flotillin 1/2 complex and counteract axonal integrity due to the function of flotillin proteins in axon growth (10, 11). Anti-flotillin-1/2 complex autoantibodies were identified in patients with multiple sclerosis (12), but until now, not in patients with severe cognitive impairment.

## Case Presentation

**Figure 1:**
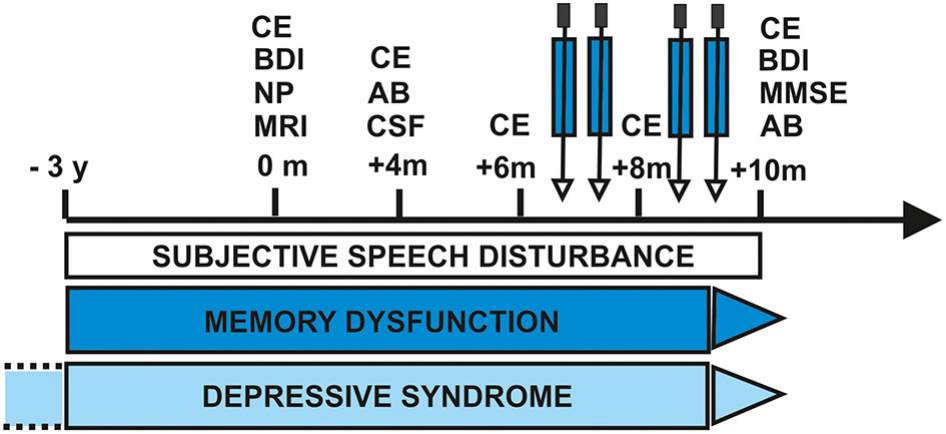
Time course of diagnostic and therapeutic approach. AB, antibody testing; BDI, Beck Depressions Inventory; CE, clinical examination; CSF, cerebrospinal fluid; m, month; MRI, magnetic resonance imaging; NP, neuropsychology; y, year. The black arrow and the arrows after the blue arrows placed after the squares with symptoms indicate an improvement of symptoms. The dotted lines before the square “depressive syndrome” indicate its repeated occurrence.

**Figure 2:**
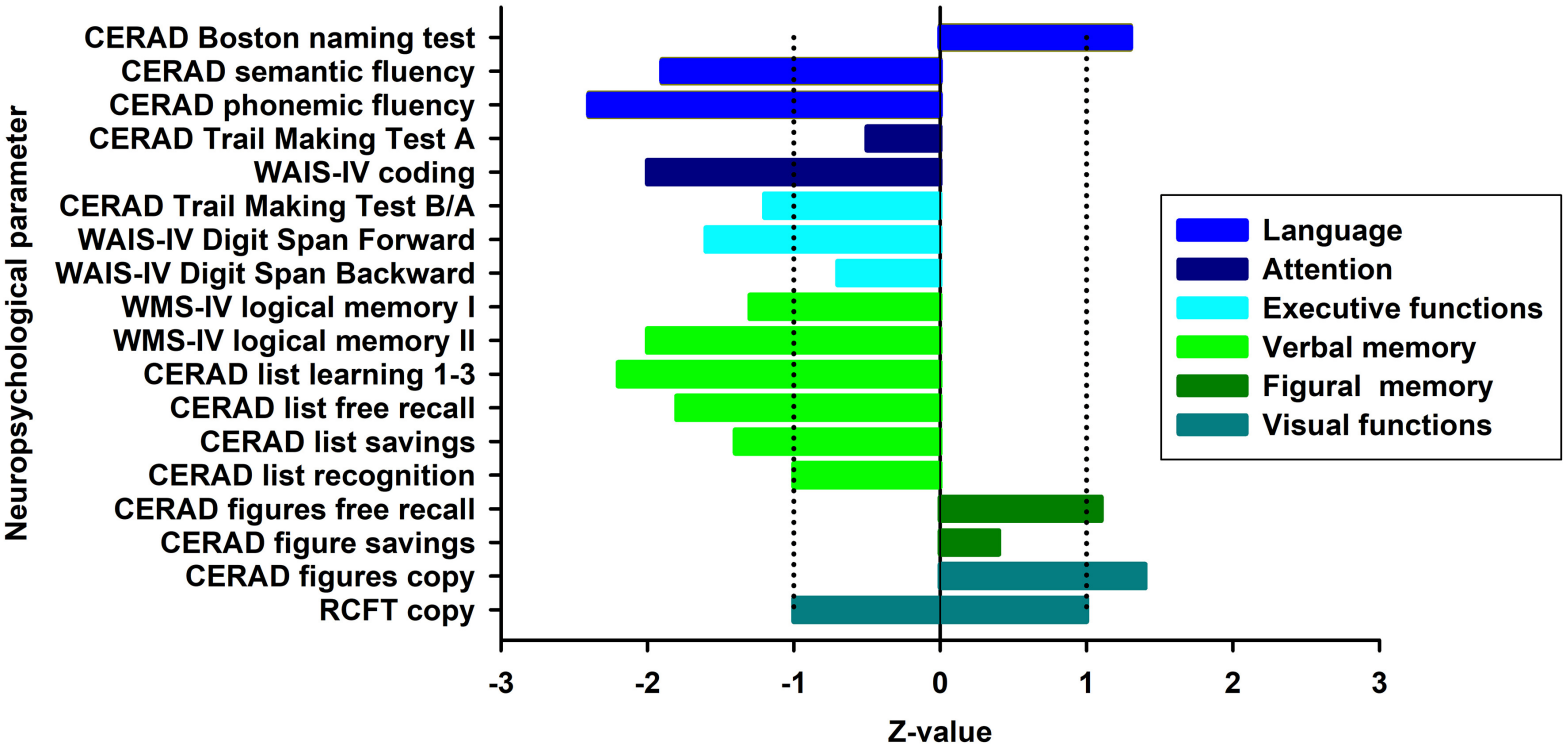
Cognitive profile at baseline. Illustration of neuropsychological test results at baseline presented as z-scores. Blue-shaded fields denote the normal range. Normative values of the RCFT Copy test only allows a distinction between a pathological and normal performance (in this case normal performance expressed as z-score between −1 and 1). The area between dotted lines indicates normal range. CERAD, Consortium to Establish a Registry for Alzheimer's Disease; WAIS-IV, Wechsler Adult Intelligence Scale—Fourth Edition; WMS-IV, Wechsler Memory Scale—Fourth Edition; RCFT, Rey Osterrieth Complex Figure Test.

At first presentation (Figure 1, first presentation) in our tertiary memory center, the patient revealed speech disturbances that had first appeared about 3 years earlier (Figure 1), starting slowly and progressing in conjunction with memory dysfunction and concentration deficits. She reported difficulty retrieving words and names. Furthermore, she reported being often stressed when having to speak, and having stagnant speech. She noticed difficulties with reading probably indicative of dyslexia and problems with comprehending speech. She also suffered from mild depressive symptoms [Beck Depression Inventory (BDI-II) score of 17] and psychomotor slowing. She is a housewife and has received 8 years of schooling. Her mother died of a heart attack and her father at 71 years of an ischemic stroke. She has two children and two grandchildren. Her psychiatric examination revealed a slightly decelerated working processing speed and the aforementioned cognition deficits. Her husband reported that she had seldom shown aggressive behavior associated with personality changes. She has been depressed, socially withdrawn, and shown little drive. Neurological examination revealed no abnormalities. Neuropsychological testing at first presentation (Figure 1) revealed impairment in verbal fluency, processing speed, cognitive flexibility, working memory span, and verbal memory (Figure 2, first presentation). The speech problems our patient had reported such as slowed speech and poorer speech comprehension were not confirmed in her neuropsychological examination at first presentation. Together with markedly impaired activities of daily living, deficits in memory, executive, and attentional functions were compatible with a diagnosis of mild dementia.

MRI at first presentation (Figure 1) revealed enlarged lateral ventricles, but no clear frontotemporal atrophy. We also observed signal hyperintensities in basal ganglia in T2 and FLAIR MRI sequences representing small vascular lesions. In both hemispheres and on the left periventricular side cerebral microangiopathy was found. CSF analysis at second presentation (Figure 1, 4 months after initial presentation) showed no pleocytosis, but elevated levels of phosphorylated tau protein 181 as a neuroaxonal destruction marker [ptau181: 65 pg/ml (pathological >61 pg/ml)]. An elevated Herpes simplex virus antibody index [2.6 (pathological >1.5)] was reported to be in agreement with a scar tissue indicating an intrathecal inflammation from an earlier infection. Furthermore, a Herpes simplex virus encephalitis was very unlikely as its typical clinical features such as fulminant onset, MRI revealing temporal lobe involvement and CSF pleocytosis are lacking. As an infectious origin of symptoms was thus improbable, we suspected an autoimmune origin of such atypical dementia in our patient. The neural autoantibodies we analyzed in serum and CSF (Figure 1) at 4 months after initial presentation via immunoblots [antibodies against paraneoplastic antigens) were glutamic acid decarboxylase 65 (GAD65), Zic4, TR, SOX1, Ma1, Ma2, Amphiphysin, CV2, Ri, Yo, and HuD] and immunofluorescence tests, as well as cell-based assays [antibodies against neural antigens such as N-methyl-D-aspartate receptor (NMDAR), leucine rich glioma inactivated protein 1 (LGI1), α-amino-3-hydroxy-5-methyl-4-isoxazolepropionic acid receptor 1 (AMPAR1), gamma aminobutyric acid B1/ 2 (GABAB1/2), AMPAR2, dipeptidyl-peptidase–like protein-6 (DPPX), contactin associated protein 2 (CASPR2), Aquaporin 4, and flotillin 1/2]. The search for flotillin 1/2 antibodies was part of the standard panel of neural autoantibodies for the differential diagnosis of atypical dementia. Serum, but not CSF immunofluorescence autoantibody testing showed anti-flotillin-1/2 autoantibodies in serum (1:32). Other laboratory tests ruled out vitamin deficiencies and metabolic disorders or infections such as lues or borreliosis as reasons for our patient's cognitive decline. We assumed an anti-flotillin 1/2-associated dementia 6 months after her first presentation (Figure 1) based on the neuropsychologically-confirmed dementia syndrome affecting several cognitive subdomains, proof of pathological tau and elevated phosphorylated tau protein in CSF, and clinical features of dementia entailing a slowly progressing onset in conjunction with obvious, subjective speech difficulties and a depressive syndrome fulfilling the criteria for autoimmune dementia according to Banks et al. (5) and Sechi and Flanagan (2). However, the depressive syndrome might, but was not proven to be a clear aspect of the disease manifestation, as the depression preceded the onset of our patient's cognitive decline by many years. Her brain MRI at first presentation was inconclusive for CNS inflammation. A limitation of our report is that we did not have her undergo further neuroimaging such as FDG PET to detect abnormal metabolism. We have considered a frontotemporal dementia, Alzheimer's dementia, and a vascular dementia as possible differential diagnoses. Multiple sclerosis has been reported coexisting with flotillin 1 antibodies (12). However, our patient's age of onset, the lack of typical cerebral lesions and of typical CSF multiple-sclerosis evidence prevented us from diagnosing MS in this case. A vascular dementia was conceivable, as that concurs with MRI findings of subcortical arteriosclerotic encephalopathy. However, we detected no time relationship between her development of cognitive symptoms and the lesions. Furthermore, the slowly progressive nature of cognitive symptoms and her neuropsychological profile argued in favor of no vasculopathy as an explanation for her cognitive decline. Alzheimer's dementia is possible, but unlikely, as we identified no amyloidopathy in CSF analysis. A frontotemporal dementia was another reasonable differential diagnosis, but the clinical criteria of FTD subtypes according to Gorno-Tempini et al. (13) were not met. As our patient's language difficulties were only one of the presenting features and were mainly subjectively reported by the patient (apart from her reduced semantic fluency, and as those deficits played no role in any impairment in her daily living capacities, we noted that the primary criteria for progressive aphasia (PPA) were not met. Thus, the variants of PPA (non-fluent, agrammatic and logopenic) were clinically not likely to explain our patient's atypical dementia. Furthermore, as none of the clinical features of the behavioral variant of frontotemporal dementia regarding disinhibition, loss of empathy, repetitive behavior or executive dysfunction were present in our case, this differential diagnosis cannot be assumed either. We had considered other tauopathies such as corticobasal degeneration and supranuclear palsy, but finally rejected these as differential diagnoses as cardinal clinical features were missing in both diseases. We therefore assumed that her dementia probably has an autoimmune-based origin, in line with recent guidelines from Banks et al. (5), which is why she was given corticosteroids starting 6 months after first presentation (Figure 1). We applied a therapeutic regimen for this autoantibody-associated psychiatric syndrome resembling autoimmune dementia, as was recently reviewed (14). We planned to give her high-dose methylprednisolone intravenously. However, as she failed to tolerate the intravenous route 3 months after diagnosis and 6 months after first presentation, two cycles were administered as a high-dosage oral therapy of 1 g prednisone per day over 3 days repeated monthly (Figure 1), planned to proceed for four further applications. Prednisone was tolerated well and no side effects appeared. The patient demonstrated therapy adherence. However, another follow-up 8 months after her first presentation failed to reveal a clear clinical benefit (this follow-up included taking her patient history and clinical examination including psychopathological and neurological examination). Two further cycles of oral steroids were given and therapy was again well-tolerated. In the clinical follow-up 10 months after initial presentation (Figure 1) and after four cycles of oral steroids, she presented objectively with less cognitive decline as assessed by mini-mental status examination (MMSE) (actual MMSE 29/30, prior MMSE 9 months earlier 27/30). She reported subjectively a partial response and slight cognitive improvement on half of the days. Furthermore, her slight cognitive improvement is accompanied by feeling less depressed on BDI (actual BDI-II value: 7, BDI-II value 9 months earlier: 17). At this second follow up, serum anti-flotilin 1/2 antibodies (1:10) were again detected in an autoantibody test in blood samples (Figure 1).

The third follow up 12 months after initial presentation including an additional cMRI, serum analysis of flotillin 1/2 autoantibodies, and neuropsychological testing including the CERAD test will be done after two more steroid cycles.

In conclusion, both our objective measurements and our patient's subjective feedback revealed an improvement in cognition and mood. Her slight clinical improvement supports the autoimmune origin of her dementia according to the guidelines on autoimmune dementia (4) and argues against a worsening neurodegenerative dementia such as FTD although markers of axonal neurodegeneration have been detected. According to Banks et al. (5), markers of axonal degeneration may be elevated in autoimmune encephalitis with cognitive dysfunction (15, 16); resembling autoimmune dementia they do not preclude the assumption of an autoimmune dementia. However, no genetic tests were conducted to exclude FTD mutations like C9 or 72 mutations. An infectious encephalitis was less likely, as it might have worsened following successful immunotherapy. Our report is in agreement with the actual version of the Declaration of Helsinki and we received informed consent for publication from our patient.

## Discussion and Conclusions

In conclusion, an autoimmune dementia (2, 5) with the autoimmune indicator of a “red flag” of a prominent reported disturbance of spontaneous speech coincided with further evidence and repeated proof of serum anti-flotillin 1/2 autoantibodies and elevated phosphorylated tau protein. Self-reported and observable mild speech and language symptoms could not be verified via neuropsychological testing, and the Gorno-Tempini et al-criteria (13) for any primary progressive aphasia of the frontotemporal dementia spectrum, including semantic dementia, have not been completely fulfilled. However, a coexisting possible Alzheimer's disease pathology cannot be fully excluded. Our patient's cognitive impairment could be caused by impaired flotillin 1 function, as flotillin 1 is a regulatory mechanism that drives presynaptic excitatory or inhibitory neuronal activity in animals (17). Blocked flotillin 1 function through anti-flotillin 1/2 autoantibodies might result in altered presynaptic neuronal transmission, possibly affecting cognition. Considering autoimmune-based dementia as experimental therapy after having disclosed test results and treatment options (2, 5, 14, 18, 19), we proposed to initiate immunotherapy with high-dose methylprednisolone. Taken together, our case reveals the novelty of serum flotillin 1/2 autoantibodies in probable autoimmune dementia, and expands the clinical spectrum that anti-flotillin 1/2 autoantibodies have exhibited so far. We further speculate that autoantibodies against flotillin 1/2 might impair the recycling (i.e., Rab5-Rab11a-dependent) of the T-cell receptors important for activating T-cells via endocytosis (20, 21). It has been hypothesized that failing T-cell activation, for instance regulatory T-cells, might lead to cell death (22) causing CNS autoimmunity. Another interesting aspect is that in the systemic lupus erythematosus autoimmune disorder, impaired flotillin-1 recruitment in lipid rafts of activated B-cells might lead to heightened disease activity, as shown by the Vasquez study (23), concurring with flotillin 1's potential hypofunctioning due to flotillin 1/2 autoantibodies. This is how anti-flotillin 1/2 autoantibodies might exacerbate brain inflammation leading to cognitive dysfunction. A hypothetical mechanism is delineated suggesting how anti-flotillin 1/2 autoantibodies might cause autoimmune processes on the one hand in step one, and on the other hand in step two, neuroaxonal degeneration marked by a high phosphorylated tau protein. This case supports existing evidence about a possible secondary neurodegeneration in autoimmune CNS inflammation (15, 16, 24). On the other hand it is conceivable, but less likely that a neurodegenerative disorder is triggered by an autoimmune process. This patient's follow up will reveal whether the first or latter hypothesis is makes more sense. The strength of this report is our novel report of an atypical dementia associated with anti-flotillin 1/2 antibodies. However, our study limitations concern the not fully evaluated immunoassay by which anti-flotillin 1/2 antibodies are detected, the lack of CSF anti-flotillin 1/2 antibodies, and the limited use of neuroimaging techniques such as no 18F-fluorodeoxyglucose positron emission tomography. We plan to conduct large-scale studies to assess the relevance and significance of this antibody in atypical dementia.

Overall, our patient's prognosis is promising, in light of recent reviews on the immunotherapeutic potential of autoantibody-associated psychiatric syndromes (19) and autoimmune dementia (2). However, we do not know if this will also apply to our patient with anti-flotillin 1/2 antibodies never before reported in dementia patients. Furthermore, considering the fact that anti-flotillin 1/2 antibodies are antibodies against membrane surface antigens, a good outcome is probable and we expect to observe further regression of symptoms, although we cannot fully exclude persisting or progressing symptoms due to existing axonal brain damage as we detected elevated phosphorylated tau protein in CSF. However, note that the repeated proof of anti-flotillin 1/2 antibodies suggests ongoing disease activity that requires further immunotherapy. Further studies in patients need to be conducted to discover whether anti-flotillin 1/2 autoantibodies are detected coinciding with specific dementia subtypes possessing a frontotemporal neurodegenerative phenotype, or if anti-flotillin 1/2 autoantibodies are characteristics of an autoimmune dementia.

## Data Availability Statement

The raw data supporting the conclusions of this article will be made available by the authors, without undue reservation.

## Ethics Statement

Ethical review and approval was not required for the study on human participants in accordance with the local legislation and institutional requirements. The patients/participants provided their written informed consent to participate in this study. Written informed consent was obtained from the individual(s) for the publication of any potentially identifiable images or data included in this article.

## Author Contributions

NH wrote the manuscript. CB, CT, JW, and WS revised the manuscript for important intellectual content. All authors contributed to the article and approved the submitted version.

## Conflict of Interest

The authors declare that the research was conducted in the absence of any commercial or financial relationships that could be construed as a potential conflict of interest.
